# Supplementary material for: Causal Relationship between Aging and Anorexia Nervosa: A White-Matter-Microstructure-Mediated Mendelian Randomization Analysis
Source: Biomedicines. 2024 Aug 16;12(8):1874. doi: 10.3390/biomedicines12081874 (PMC11351342; doi:10.3390/biomedicines12081874)
Supplement: Supplementary file 1 [file biomedicines-12-01874-s001.zip › Supplementary Figure/Figure S8/Leaveoneout plot of MR analysis for TL to ubm-b-1835.pdf]

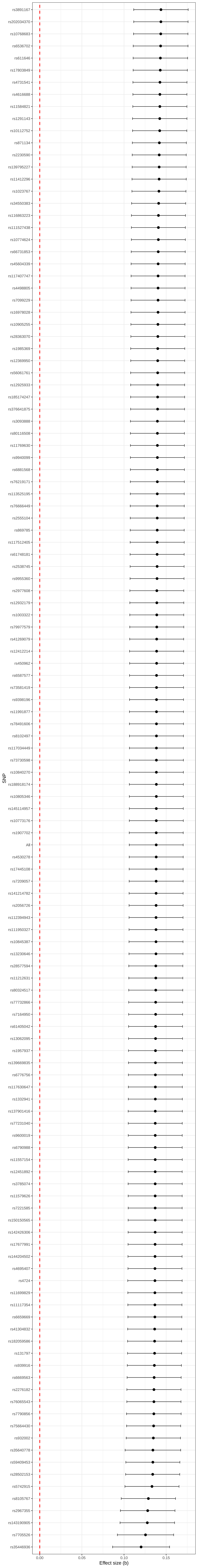
rs3891167
rs202034370
rs10768683
rs6536702
rs611646
rs17803849
rs4731541
rs4616688
rs11584821
rs1291143
rs10112752
rs871134
rs2230590
rs139795227
rs11412296
rs1023767
rs34550383
rs116863223
rs111527438
rs10774624
rs66731853
rs45604339
rs117407747
rs4498805
rs7099229
rs16978028
rs10905255
rs28363070
rs1985369
rs12369950
rs56061761
rs12925933
rs185174247
rs376641875
rs3093888
rs80116508
rs11769630
rs9940099
rs6881568
rs76219171
rs113525195
rs76666449
rs2555104
rs869785
rs117512405
rs61748181
rs2538745
rs9955360
rs2977608
rs12932179
rs1003322
rs79977579
rs41269079
rs12412214
rs450962
rs6587577
rs73581419
rs9398196
rs11991877
rs78491606
rs8102497
rs117034449
rs73730598
rs10840270
rs188918174
rs10805346
rs145114957
rs10773176
rs1907702
All
rs4530278
rs17445108
rs7209057
rs141214782
rs2056726
rs112394943
rs111950327
rs10845387
rs13230646
rs28577594
rs11212631
rs80324517
rs77732866
rs7164950
rs61405042
rs13062095
rs1957937
rs139669835
rs6776756
rs117630647
rs1332941
rs137901416
rs77231040
rs9600019
rs6790988
rs11557154
rs12451892
rs3785074
rs11579626
rs7221585
rs150150565
rs142426306
rs17677991
rs144204502
rs4695407
rs4724
rs11699829
rs11117354
rs6659669
rs41304832
rs182059586
rs131797
rs939916
rs6669563
rs2276182
rs76065543
rs7790856
rs75664430
rs932002
rs35640778
rs59409453
rs28502153
rs5742915
rs8105767
rs2967355
rs143190905
rs7705526
rs35446936
SNP
0.00
0.05
0.10
0.15
Effect size (b)
